# Effect of human interferon-λ1 recombinant adenovirus on a gastric cancer orthotopic transplantation model

**DOI:** 10.3892/etm.2014.1896

**Published:** 2014-08-11

**Authors:** XU-FENG BU, JIE ZHANG, LI-JUAN JIA, BING LIANG, JIN ZHANG, YANG LIU, YU-LAN YAN

**Affiliations:** 1 Department of General Surgery, Affiliated People’s Hospital of Jiangsu University, Zhenjiang, Jiangsu 212002, P.R. China; 2 Clinical Medicine College of Jiangsu University, Zhenjiang, Jiangsu 212002, P.R. China; 3 Department of Internal Medicine, Affiliated People’s Hospital of Jiangsu University, Zhenjiang, Jiangsu 212002, P.R. China

**Keywords:** human interferon-λ1 recombinant adenovirus, gastric cancer, orthotopic transplantation, apoptosis

## Abstract

The aim of the present study was to investigate the effect of human interferon-λ1 recombinant adenovirus (r-Ad-hIFN-λ1) on gastric carcinoma. Human SGC-7901 cells were utilized to create an orthotopic implantation model of gastric cancer in nude mice through sterile surgery. The mice were randomly divided into three groups: Phosphate-buffered saline control (blank), adenovirus encoding bacterial β-galactosidase (Ad-Lac Z) empty vector and r-Ad-hIFN-λ1. Tumor size was measured every seven days. After three weeks of treatment, the tumors in the mice were detected by abdominal B ultrasound. The cDNA of IFN-λ1 expression in skeletal muscle was detected by a reverse transcription polymerase chain reaction and IFN-λ1 protein expression in the tumors was detected by western blot analysis and immunohistochemistry. Flow cytometry and terminal deoxynucleotidyl transferase-mediated dUTP-biotin nick end labeling (TUNEL) assays were conducted to analyze the proportion of natural killer (NK) cells in the spleen and the rate of cell apoptosis in tumor paraffin sections. Prior to sacrifice, the size of the tumors in the r-Ad-hIFN-λ1, Ad-Lac Z and blank groups was 184.29±10.84 mm^3^, 234.62±10.59 mm^3^ and 253.18±7.69 mm^3^, respectively (P<0.001). The lymph node metastasis in the abdominal cavity was 0% in the r-Ad-hIFN-λ1 group, 50% in the Ad-Lac Z group and 80% in the blank group (P<0.005). Furthermore, IFN-λ1 mRNA and protein were highly expressed in the r-Ad-hIFN-λ1 group, and the apoptosis rate in the r-Ad-hIFN-λ1 group was higher than that in the Ad-Lac Z and blank groups. The proportion of NK cells in the spleens of nude mice in the r-Ad-hIFN-λ1, Ad-Lac Z and blank groups was 26.53±1.54, 17.70±1.09 and 16.35±1.43%, respectively (P<0.001). The TUNEL results showed there was significantly more severe apoptosis in the r-Ad-hIFN-λ1 group than that in the two other groups. The apoptosis indices in the r-Ad-hIFN-λ1, Ad-Lac Z and blank groups were 0.772±0.075, 0.329±0.169 and 0.265±0.049, respectively. In conclusion, the r-Ad-hIFN-λ1 significantly inhibited human gastric cancer, possibly by promoting apoptosis of the tumors and stimulating immunological function.

## Introduction

In 2003, Sheppard *et al* (1) identified a new family of interferon (IFN), which was named the IFN-λ family (IFN-λ1, -λ2 and -λ3) by Kotenko *et al* (2). The Human Genome Organization also temporarily provided the names interleukin (IL)-28A, IL-28B and IL-29 (1). IFN-λs utilize different receptor complexes for signal transduction from the classical type I IFNs; however, the biological effects and even the activation of the intracellular signal pathways are similar. Although using distinct receptor complexes, IFN-λs appear to induce similar intracellular signals to type I IFNs, upsetting cell apoptosis and inhibiting cell proliferation through the activation of caspase-3, caspase-8 and caspase-9 (3). The antiviral and immunomodulatory activity of IFN-λs has previously been demonstrated (4). Quantitative analysis of IFN-λ expression in human immune cell populations has revealed a particularly strong expression in T and natural killer (NK) cells (5), and it has been found that, similar to human immune B, T and NK cells, human liver cells express IL-28 receptor 1 (IL-28R1) and accept IFN-λ1, -λ2 and -λ3. Furthermore, human keratinocytes, intestinal epithelial cells, the A549 lung adenocarcinoma cell line, HepG2 and HU7 human hepatoma cells and human esophageal cancer cells express IFN-λ1, -λ2 and -λ3 (6). IFN-λ1, -λ2 and -λ3 exhibit a direct anti-proliferative activity, and the human IL-28 plasmid vector can significantly inhibit murine tumor growth (7).

It has been confirmed that a desired gene carried by an adenoviral vector can be stably expressed in a host cell (8); adenoviral DNA cannot be integrated into the host chromosome and thus poses no risk. Furthermore, adenoviruses exhibit a broad spectrum of cell invasion and their use is associated with fewer restrictions, thereby making them ideal viral vectors. The purpose of this study was to identify the anti-proliferative and immunomodulatory effects of human IFN-λ1 recombinant adenovirus (r-Ad-hIFN-λ1) on gastric cancer *in vivo* in a nude mouse model of human gastric cancer.

## Materials and methods

The recombinant adenoviruses r-Ad-hIFN-λ1 and adenovirus encoding bacterial β-galactosidase (Ad-Lac Z) were successfully constructed by Mr. Yang Liu (Affiliated People’s Hospital of Jiangsu University, Zhenjiang, China) and stored at the College of Clinical Medicine of Jiangsu University (Zhenjiang, China) (9). The SGC-7901 cell line was obtained from the Cell Culture Center of the Basic Institute of Medical Sciences, Peking Union Medical College (Beijing, China). Four-week-old BALB/c (nu/nu) mice, weighing 14–16 g, were provided by the Comparative Medicine Center of Yangzhou University (Yangzhou, China) and were raised in the Experimental Animal Center of Jiangsu University. FuAiLe (FAL) medical glue was obtained from the Beijing Institute of Medical Glue (Beijing, China). TRIzol^®^ reagent and anti-IFN-λ1 antibody were purchased from Invitrogen Life Technologies (Carlsbad, CA, USA) and Santa Cruz Biotechnology, Inc. (Santa Cruz, CA, USA), respectively. Immunohistochemistry-related reagents were purchased in Gene Tech Biotechnology (Shanghai) Co., Ltd. (Shanghai, China). All polymerase chain reaction (PCR) primers were synthesized by Shanghai Sangon Biological Engineering Technology & Services Co., Ltd. (Shanghai, China). Fluorescein isothiocyanate (FITC)-anti-mouse cluster of differentiation (CD)49b and -CD3 were purchased from eBioscience, Inc. (San Diego, CA, USA). The terminal deoxynucleotidyl transferase-mediated dUTP-biotin nick end labeling (TUNEL) assay kit was obtained from Nanjing KGI Biotechnology Development Co., Ltd. (Nanjing, China). Dulbecco’s modified Eagle’s medium (DMEM) and fetal bovine serum (FBS) were purchased from Gibco-BRL (Grand Island, NY, USA).

### Construction of the human gastric cancer orthotopic transplantation model

The SGC adenocarcinoma cells were cultured in DMEM with 10% FBS and incubated at 37°C and 5% CO_2_. SGC-7901 cells were regulated to a cell number of 1×10^6^/ml, and 200 μl was subcutaneously inoculated at the back of the right forelimb in the nude mice with a 1-ml syringe. When the diameter of the subcutaneous tumor increased to 1 cm, the tumor was removed into another nude mouse, a procedure that was repeated three times. In brief, the subcutaneous tumor-bearing nude mice were sacrificed by anesthesia overdose and the tumors were removed and sterilely cut into ~l-mm^3^ sections. New nude mice were then anesthetized with ketamine (70 μg/kg), an incision was made in the abdominal wall, exposing the stomach serosa, and gentle scarification was performed. Tumor tissue blocks were fixed in the greater curvature of the stomach with FAL surgical medical glue, and the stomach was then returned to the abdominal cavity. The abdominal incision was closed layer-by-layer, with erythromycin ointment applied for protection.

### Animal experiments

Following division into three groups, the orthotopic mouse models of human gastric cancer received injections in the skeletal muscle of the right leg as follows: Blank group, phosphate-buffered saline (PBS; 2 ml); Ad-Lac Z group, 4×10^8^ pfu Ad-Lac Z (2 ml); and r-Ad-hIFN-λ1 group, 4×10^8^ pfu r-Ad-hIFN-λ1 (2 ml). The injection was performed once a week for three weeks. The volume of the tumor was measured on days 0, 7, 14 and 21. All nude mice were sacrificed after 28 days, and the adenoma, skeletal muscle, spleen and liver were dissected *in situ.* A quantity of each tissue was fixed with 4% paraformaldehyde and the remaining tissue was stored at −80°C.

### Tumor growth curve and inhibition rates

Once the gastric cancer orthotopic model had been established for two weeks, the short and long diameters of the tumors were measured using a vernier caliper every seven days. Prior to being sacrificed, the nude mice underwent an abdominal B ultrasound. The volumes of the tumors were calculated using the following formula: V=a^2^ × b × 0.52, where ‘V’ refers to tumor volume, and ‘a’ and ‘b’ refer to the short and long diameters of the tumor, respectively. The tumor inhibition rates were calculated according to the last measurement of tumor volume with the following computational formula: Inhibition rate = (average tumor volume of the blank group - average tumor volume of the r-Ad-hIFN-λ1 group)/average tumor volume of the blank group × 100%.

### Histopathology analysis

The orthotopically transplanted tumor and the liver and enlarged lymph nodes were fixed with 4% paraformaldehyde. Tissue blocks were dehydrated, cleared, paraffin-embedded and sectioned at 5-μm thickness. The sections were stained by a hematoxylin and eosin (HE) staining procedure. Pathological changes were observed with an optical microscope (Axio Observer A1m; Pury Seth Instrument Co., Ltd., Beijing, China) and images were captured.

### Immunohistochemistry

The tissue sections were prepared at 60°C overnight. The paraffin sections were rinsed in xylene for 10 min twice and dewaxed using an ethanol gradient (100, 95, 90, 80 and 70%), prior to undergoing antigen retrieval in boiling sodium citrate-hydrochloric acid buffer solution for 20 min. The sections were then washed with PBS three times for 5 min each, incubated with anti-IFN-λ1 antibody (dilution ratio 1:100) at 4°C overnight, washed with PBS for 5 min three times and incubated with anti-rabbit antibody (GT Vision™ I type polymer) for 10 min. A further three 5-min washes with PBS were then performed, prior to the section being incubated with 3,3′-diaminobenzidine (DAB) working solution for 10 min, washed with PBS for 5 min three times and stained for 5 sec with hematoxylin. The sections were subsequently further washed with PBS for 5 min three times, dehydrated using an ethanol gradient (70, 80, 90, 95 and 100%), and then rinsed in xylene for 10 min twice. A neutral rubber seal was subsequently applied.

### Reverse transcription (RT)-PCR

Skeletal muscle tissues were cut into 50-mg sections and homogenized on ice. Following centrifugation at 16,000 × g for 20 min at 4°C, the supernatant was discarded. The total RNA was extracted from the pellet by TRIzol^®^ reagent. The cDNA was synthesized with Oligo (dT) primers and moloney murine leukemia virus reverse transcriptase. One-tenth of the cDNA was used for PCR amplification and the PCR products were separated in 1% agarose gels by electrophoresis. The PCR protocol was as follows: Initial denaturation for 5 min at 94°C, followed by 30 cycles for 30 sec at 94°C, annealing (IFN-λ1) for 30 sec at 53°C and extension for 30 sec at 72°C. The final extension was performed by an incubation step for 10 min at 72°C. The PCR products were subjected to electrophoresis in 1% agarose gel and visualized by ethidium bromide. The bands were analyzed using Quantity One^®^ software (Bio-Rad, Hercules, CA, USA). All the primers are listed in Table I.

### Western blotting

The IFN-λ1 protein was detected by western blotting. Tumor tissue (1 g) was cut into 1×1×1 mm sections and homogenized on ice. Following rapid centrifugation at 224 × g, the supernatant was discarded and the pellet was resuspended with 1,000 μl pre-cooled radioimmunoprecipitation assay lysis buffer, consisting of 3 μl sodium orthovanadate, 3 μl phenylmethyl sulfonyl fluoride and 3 μl protease inhibitor cocktail. The mixture was homogenized and lysed for 60 min on ice. Following centrifugation at 12,000 × g for 15 min at 4°C, the supernatant was transferred into another 1.5-ml tube and mixed with an equal volume of loading buffer (2×) and β-mercaptoethanol (20×). The tubes were then placed in boiling water for 10 min. The extracted protein was stored at −80°C. Extracted lysates (5 μl) were separated using 12% SDS-PAGE and the protein expression of IFN-λ1 was confirmed by western blot analysis with the specific antibody.

### Apoptosis assay

The apoptosis assay was performed using the TUNEL kit according to the manufacturer’s instructions (Nanjing KGI Biotechnology Development Co., Ltd.). Briefly, the paraffin sections were prepared overnight at 60°C, rinsed in xylene for 10 min and then immersed in the graded ethanol series. Subsequent to washing with PBS for 5 min twice, the samples were each incubated with proteinase K work liquid (100 μl) for 30 min at 37°C and washed with PBS for 5 min three times. The samples were then incubated with 3% H_2_O_2_ methanol for 10 min at room temperature (15–25°C) and washed with PBS for 5 min three times. The positive and negative controls were set up. Pre-treated specimens were incubated with 50 μl terminal deoxynucleotidyl transferase labeling reaction buffer for 60 min at 37°C in the dark. Subsequently, the specimens were incubated with 50 μl streptavidin-horseradish peroxidase for 30 min in the dark and 50 μl DAB coloration solution was applied for 5 min. The sections were observed and images were captured using an optical microscope. Under a magnification of ×400, areas were selected where the apoptotic cells were distributed evenly, and the number of positive cells out of 100 cells was counted. This was repeated three times. The apoptosis index (AI) was calculated using the following formula: AI = apoptotic cell number/(apoptotic cell number + non-apoptotic cell number) × 100%.

### Flow cytometry

Flow cytometry was performed to detect the number of NK cells in the spleen. Subsequent to sacrifice, the spleen of each mouse was gently removed and made into a splenocyte suspension. The erythrocytes were lysed by the addition of 0.83% Tris-NH_4_Cl solution. Following rinsing with PBS (pH 7.4) three times, the splenocytes were re-suspended with staining buffer at a density of 1×10^6^/ml. The splenocytes were dispensed into 100 μl aliquots, placed into tubes and surface-labeled with 5 μl hamster anti-CD3e-phycoerythrin and CD49b-FITC (the NK cells were CD3^−^ and CD49^+^). The mixture was incubated at 4°C for 30 min in the dark, prior to the addition of 200 μl pre-cooled staining buffer. Following centrifugation at 126 × g for 10 min, the precipitates were re-suspended twice and fixed in 200 μl 1% paraformaldehyde. Labeled cells were washed and analyzed with a FACSCalibur™ flow cytometer (BD Biosciences, Bedford, MA, USA) by Windows Multiple Document Interface for Flow Cytometry 2.9 software (Microsoft Corp., Redmond, WA, USA) and CellQuest™ software (BD Biosciences). In each sample, staining was compared with that of an appropriately labeled isotype control antibody.

### Statistical analysis

The results were analyzed with Statistical Analysis System (SAS) 6.12 (SAS Institute Inc., Cary, NC, USA) and are expressed as the mean ± standard deviation. To compare the differences among the groups, statistical significance was analyzed using Pearson’s χ^2^ test and a one-way analysis of variance followed by post hoc comparisons. P<0.05 was considered to indicate a statistically significant difference.

## Results

### Tumor growth curve and inhibition rates

**Figure 1:**
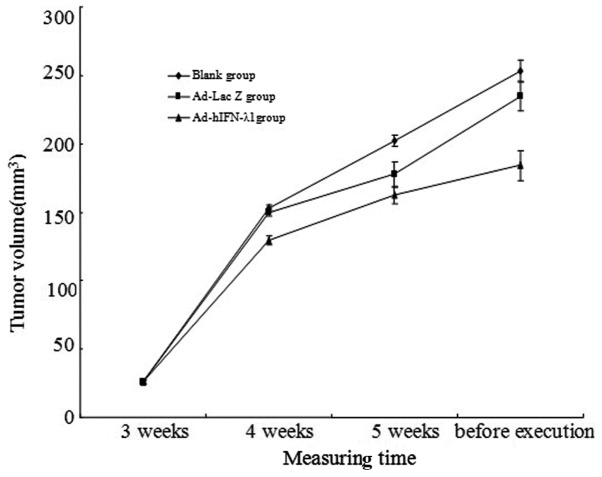
Curve of tumor growth. r-Ad-hIFN-λ1, human interferon-λ1 recombinant adenovirus; Ad-Lac Z, adenovirus encoding bacterial β-galactosidase.

**Figure 2:**
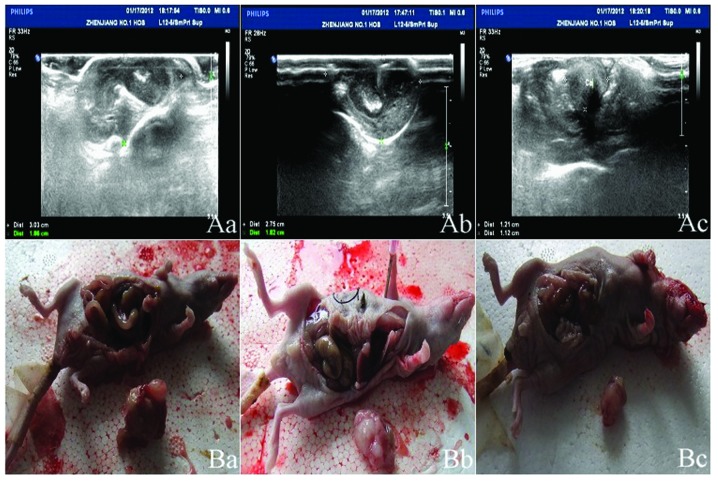
(A) B ultrasound of the abdomen prior to sacrifice and (B) status of the tumor growth of the three groups. (Aa and Ba) Blank, (Ab and Bb) Ad-Lac Z and (Ac and Bc) r-Ad-hIFN-λ1 groups. Following a month of intervention the tumor volume of the r-Ad-hIFN-λ1 group was markedly lower than that of the blank and Ad-Lac Z groups. Ad-Lac Z, adenovirus encoding bacterial β-galactosidase; r-Ad-hIFN-λ1, human interferon-λ1 recombinant adenovirus.

Tumor volumes notably increased in the blank and Ad-Lac Z groups during the study. Although slight increases in tumor volumes were also present in the r-Ad-hIFN-λ1 group, the volumes were significantly lower than those in the blank and Ad-Lac Z groups (Fig. 1 and Table II). The abdominal B ultrasound showed the size and position of the orthotopically transplanted tumors (Fig. 2). The inhibition rate in the r-Ad-hIFN-λ1 group was 27.21% [(253.18-184.29)/253.18×100%]. The abdominal laparotomy revealed that following a month of intervention, the r-Ad-hIFN-λ1 group exhibited no lymph node metastasis, whereas the Ad-Lac Z and blank groups exhibited high levels of lymph node metastasis (Table III).

### Morphological analysis

**Figure 3:**
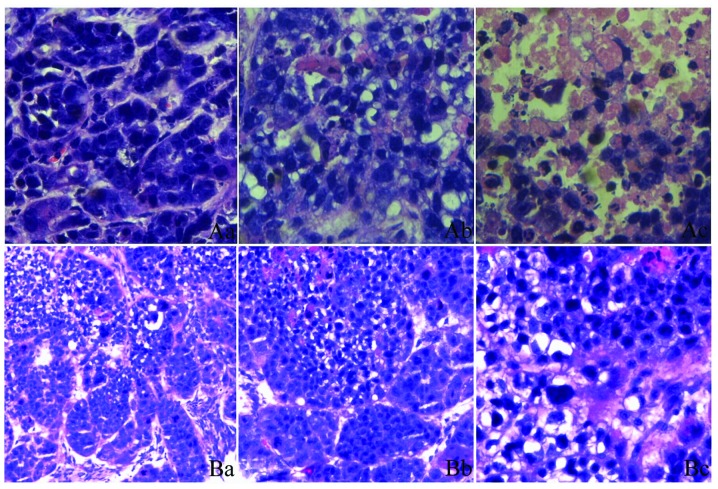
Hematoxylin and eosin staining of (A) orthotopic transplantation tumor tissue sections and (B) intra-abdominal intumescent lymph nodes. (Aa-c) Orthotopic transplantation tumor tissue sections in the (Aa) blank, (Ab) Ad-Lac Z and (Ac) r-Ad-hIFN-λ1 groups. Magnification, ×200. (Aa) Blank: Cells with large and deeply stained nuclei, which replaced normal glandular tissue. (Ab) Ad-Lac Z: Presence of a little necrosis. (Ac) r-Ad-hIFN-λ1: Presence of considerable necrosis. (Ba-c) Intra-abdominal intumescent lymph nodes at magnifications of (Ba) ×100, (Bb) ×200 and (Bc) ×400. Ad-Lac Z, adenovirus encoding bacterial β-galactosidase; r-Ad-hIFN-λ1, human interferon-λ1 recombinant adenovirus.

HE staining of the orthotopic transplantation tumor tissue sections showed that a number of cells exhibited a circular disordered arrangement and had large and deeply stained nuclei. These cells replaced the normal glandular tissue. Intra-abdominal intumescent lymph nodes were found in the blank and Ad-Lac Z groups. The HE staining revealed the normal structure of the lymph nodes to be damaged and replaced by invasive tumor cells similar to the orthotopic transplantation tumor cells in the histomorphology (Fig. 3).

### Immunohistochemistry

**Figure 4:**
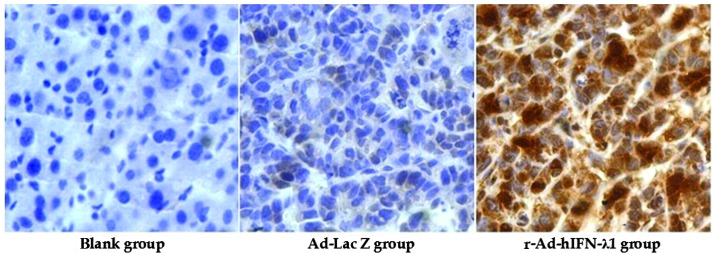
Immunohistochemistry for IFN-λ1 protein. IFN-λ1 protein was strongly expressed in the cytoplasm of the r-Ad-hIFN-λ1 group but showed little expression in the other two groups. r-Ad-hIFN-λ1, human interferon-λ1 recombinant adenovirus; Ad-Lac Z, adenovirus encoding bacterial β-galactosidase. Magnification, ×200.

The protein expression of IFN-λ1 in the tumor tissue was detected by immunohistochemistry. Strongly positive IFN-λ1 protein expression was observed in the cytoplasm in the r-Ad-hIFN-λ1 group; however, the other two groups were negative for IFN-λ1 protein expression. This indicated that IFN-λ1 was strongly expressed in the tumor cells in the r-Ad-hIFN-λ1 group (Fig. 4).

### Expression of hIFN-λ1 mRNA

**Figure 5:**
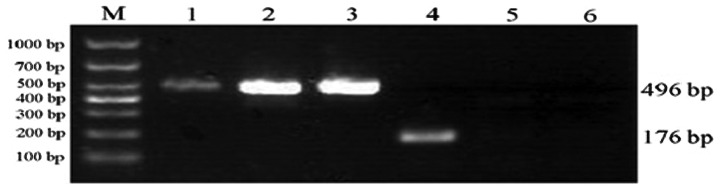
IFN-λ1 mRNA expression in skeletal muscle. The r-Ad-hIFN-λ1 group expressed IFN-λ1 mRNA, while no expression was identified in the other two groups. Lane: M, Marker; 1, GAPDH (r-Ad-hIFN-λ1 group); 2, GAPDH (Ad-Lac Z group); 3, GAPDH (blank group); 4, hIFN-λ1 (r-Ad-hIFN-λ1 group); 5, hIFN-λ1 (Ad-Lac Z group); 6, hIFN-λ1 (blank group). r-Ad-hIFN-λ1, human interferon-λ1 recombinant adenovirus; Ad-Lac Z, adenovirus encoding bacterial β-galactosidase.

RT-PCR was performed to confirm the transfection of hIFN-λ1 mRNA in the skeletal muscle. The analysis showed evident expression of hIFN-λ1 mRNA (~176 bp) following infection by r-Ad-hIFN-λ1. In the blank and Ad-Lac Z groups, no hIFN-λ1 mRNA expression was observed (Fig. 5).

### Protein expression of IFN-λ1

**Figure 6:**
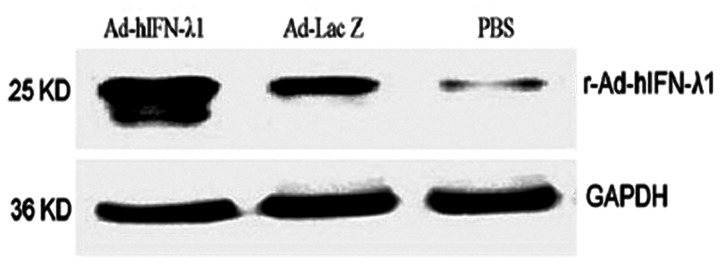
hIFN-λ1 protein expression. Expression was strong in the r-Ad-hIFN-λ1 group, weak in the Ad-Lac Z group and minimal in the blank (PBS) group. r-Ad-hIFN-λ1, human interferon-λ1 recombinant adenovirus; Ad-Lac Z, adenovirus encoding bacterial β-galactosidase; PBS, phosphate-buffered saline.

Western blotting was performed to confirm the expression of hIFN-λ1 protein in the tumor tissues. The results showed notable hIFN-λ1 expression in the tumor tissues following infection by r-Ad-hIFN-λ1. In the Ad-Lac Z group, low levels of hIFN-λ1 were expressed. In the blank group, hIFN-λ1 expression was minimal (Fig. 6)

### Apoptosis of tumor cells

**Figure 7:**
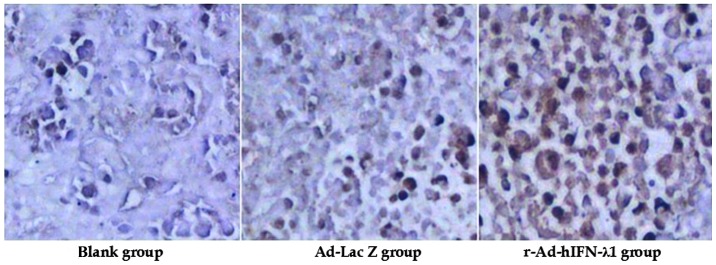
Apoptosis in the nude mice tumor paraffin sections in the three groups as revealed by terminal deoxynucleotidyl transferase-mediated dUTP-biotin nick end labeling. The number of apoptotic cells in the r-Ad-hIFN-λ1 group was significantly higher than that in the other two groups. r-Ad-hIFN-λ1, human interferon-λ1 recombinant adenovirus; Ad-Lac Z, adenovirus encoding bacterial β-galactosidase. Magnification, ×200.

TUNEL assay was performed to determine the level of apoptosis in the tumor tissue paraffin sections. As expected, the AI was significantly higher in the r-Ad-hIFN-λ1 group than that in the blank and Ad-Lac Z groups. No significant difference was identified between the blank and Ad-Lac Z groups (Fig. 7 and Table IV).

### Flow cytometry

**Figure 8:**
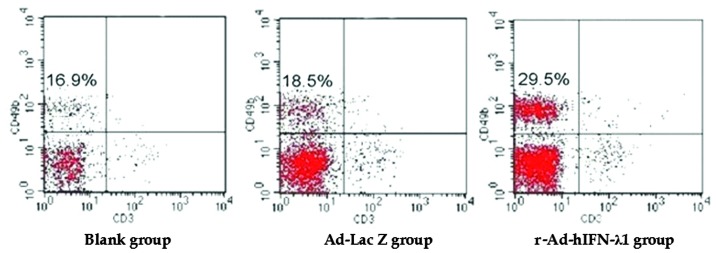
Detection of natural killer cell proliferation in the spleen. Proliferation occurred at a higher rate in the r-Ad-hIFN-λ1 group than in the other two groups. r-Ad-hIFN-λ1, human interferon-λ1 recombinant adenovirus; Ad-Lac Z, adenovirus encoding bacterial β-galactosidase; CD, cluster of differentiation.

Flow cytometry was performed to detect the number of NK cells in the spleen. The results showed that the number of NK cells was significantly higher in the r-Ad-hIFN-λ1 group than that in the blank and Ad-Lac Z groups. No significant difference was identified between the blank and Ad-Lac Z groups (Fig. 8 and Table V).

## Discussion

It has been shown that the IFN family exhibits a significant anti-tumor effect (4). Thus, IFN is widely clinically applied as a biological therapy to target hairy cell leukemia, chronic myelogenous leukemia, renal cell carcinoma and melanoma (10). IFN was also the first cytokine to undergo complete sequencing, be purified to homogeneity, cloned, produced in a recombinant form and used extensively in clinical application (11). However, treatment with type I IFNs can occasionally cause significant side-effects, including fatigue, fever, anorexia, depression and myelosuppression. When prolonged, for instance in the case of hepatitis C treatment, type I IFN treatment can lead to neurological or neuropsychiatric adverse effects (12). Since the receptor for IFN-λ exhibits a restricted pattern of expression, IFN-λ may serve as a therapeutic alternative to type I IFNs and reduce the adverse side-effects associated with type I IFN therapy (13).

At present, IFN-λs are a relatively novel member of the cell factor family administered in tumor treatment. It has been shown that IFN-λs inhibit a wide variety of tumors and induce their apoptosis to the same degree as the type I IFNs (4). IFN-λs are different from type I IFNs in their structure and respective receptors. The type I IFN receptors are encoded by IFNα receptor (IFNAR) 1 and IFNAR2; by contrast, IFN-λs belong to the type III group of IFNs, the receptor complexes of which exhibit a restricted pattern of expression. The receptors of type III IFNs have been identified as IL-28Rα and IL-10Rβ; IL-10Rβ has also been shown to be one of the receptor compounds of IL-10 and IL-10-related IL receptors, such as IL-22 and IL-26 (14). Although type I and type III IFNs signal through distinct receptors, they both induce anti-proliferative responses. The anti-proliferative activity of IFN-λ1 has been previously demonstrated in a subclone of the murine BW5147 thymoma cell line transfected with human IFN-λR (15). Due to the differences between the two types of IFN receptor complexes, it could be hypothesized that the cells expressing different receptor complexes may exhibit different reactions to IFN-λs and type I IFNs. A previous study showed that the type I IFN receptor exists on the surface of almost all cells and that the distribution of IL-10Rβ is extensive. However, the expression of IL-28Rα appears to be limited to specific tissues, with almost no expression in fibroblasts and endothelial cells (16). In the present study, western blotting and the immunohistochemical study of the tumors of the r-Ad-hIFN-λ1 group showed that IFN-λ1 was successfully transfected and effectively expressed in the cytoplasm of tumor cells injected into skeletal muscle. This suggested that the IFN-λ1 receptor was expressed on the surface of the gastric carcinoma cells.

In addition to antiviral effects, IFN-λs also exhibit immunomodulatory effects that overlap with those of type I IFNs in the innate and adaptive arms of the immune system. It has been established that type I IFNs have the effect of stimulating immune function, mainly by enhancing the response of T helper (Th) 1 cells and thereby increasing the amount of major histocompatibility complex-I and the NK and T-cell-mediated killing effect. Although the mechanism underlying the effect of IFN-λs on the immune system was unclear, previous studies showed that IFN-λ induced an increase in the levels of Th2 cells in peripheral blood (17) and induced innate and adaptive immune responses against tumors (18). IFN-λs modulate the immune system in a similar manner to the type I IFNs; however, IFN-λs additionally exert an anti-angiogenesis effect (19). In the present study, flow cytometry was used to detect the number of NK cells in the spleen blood in the r-Ad-hIFN-λ1 group. The immunoregulatory effects of IFN-λ are cell type-specific, depending on the distribution of IFN-λRs, the nature of the signal transduction and the genes activated. Furthermore, IFN-λs are capable of signaling through almost all signal transducer and activator of transcription molecules, therefore exhibiting broader functions than the type I IFNs (20).

Type I IFNs exhibit an anti-proliferative effect that is predominantly caused by apoptosis and stagnation of the cell cycle. The effect has been suggested to be associated with the tumor suppressor gene p53, as shown by IFN-mediated apoptosis in tumor cells with p53 mutation (21). Of note, Sato *et al* (7) showed that IFN-λ induced tumor apoptosis and NK cell-mediated tumor destruction through innate immune responses. Furthermore, IFN-λ expression in tumor cell lines markedly inhibited subcutaneous and metastatic tumor formation *in vivo*. A study using rats also found that IFN-λs exhibited certain inhibitory effects on tumor metastasis by regulating neutrophil, NK and CD8^+^ T cells (19). A previous *in vivo* study on melanoma in nude mice confirmed that IFN-λs inhibited the growth of the melanoma by stimulating autologous action that resulted in tumor death (22). However, Numasaki *et al* (18) conducted an *in vitro* study administering an IFN-λ intervention to a fibrosarcoma MCA205 cell line, and revealed that the multicore-shape white blood cells, neutrophils, NK cells and CD8^+^ cells played an important role in the inhibition of the tumor cell growth process. In the present study, the pathophysiological process of gastric cancer was successfully simulated in mice.

In the present study, the tumor volume of the r-Ad-hIFN-λ1 group was significantly lower than that of the blank or Ad-Lac Z groups, and no intra-abdominal intumescent lymph nodes were observed in the r-Ad-hIFN-λ1 group. The analysis of tumor paraffin sections by TUNEL showed a higher level of apoptosis in the r-Ad-hIFN-λ1 group than in the other two groups*.* These findings indicated that r-Ad-hIFN-λ1 effectively inhibited the growth and metastasis of gastric cancer and promoted gastric cancer cell apoptosis.

In conclusion, IFN-λ1 was capable of inhibiting tumor growth, decreasing the rate of tumor metastasis, as well as killing tumor cells directly or indirectly. At the same time, IFN-λ1 stimulated the immune system, leading to an indirect anti-tumor effect. In future studies we aim to alter the structure or administration route of IFN-λ1 in order to improve accordance with clinical anti-tumor biological agents.

## Figures and Tables

**Table I:** Primers for quantitative polymerase chain reaction amplification.

Primer	Sequence	Product size, bp
IFN-λ1		
Upstream	5′-TATCCAGCCTCAGCCCACAGCA-3′	176
Downstream	5′-ACAGGTTCCCATCGGCCACATA-3′	
β-actin		
Upstream	5′-CACGAAACTACCTTCAACTCC-3′	262
Downstream	5′-ACAGGTTCCCATCGGCCACATA-3′	

IFN, interferon.

**Table II:** Growth of tumor and comparison of tumor volume among the three groups.

	Group				
			
Measurement time	Blank (mm^3^)	Ad-Lac Z (mm^3^)	r-Ad-hIFN-λ1 (mm^3^)	F-value	P-value
7 days	26.06±1.15	25.72±1.50	25.70±1.11	0.256	0.776
14 days	152.68±2.51	149.62±2.41	129.19±3.30^a^	212.64	<0.001
21 days	202.18±4.31	177.62±8.88	162.19±6.38^b^	88.251	<0.001
Prior to sacrifice	253.18±7.69	234.62±10.59	184.29±10.84^b^	131.90	<0.001

a r-Ad-hIFN-λ1 group versus the other two groups, P<0.001;

b r-Ad-hIFN-λ1 group versus the Ad-Lac Z group, P<0.001.

r-Ad-hIFN-λ1, human interferon-λ1 recombinant adenovirus; Ad-Lac Z, adenovirus encoding bacterial β-galactosidase.

**Table III:** Abdominal lymph node metastasis rate.

Group	Abdominal lymph node metastasis		Total (n)	Metastasis rate (%)

	+ (n)	− (n)		
Blank	8	2	10	80
Ad-Lac Z	5	5	10	50
r-Ad-hIFN-λ1	0	10	10	0
Total	13	17	30	46.7

χ^2^=13.30; degrees of freedom, 2. P<0.005, evaluated by the boundary value table of χ^2^. r-Ad-hIFN-λ1, human interferon-λ1 recombinant adenovirus; Ad-Lac Z, adenovirus encoding bacterial β-galactosidase; +, positive; -, negative.

**Table IV:** Comparison of the AI of apoptotic cells among the three groups.

Group	AI (n=10)	F-value	P-value
Blank	0.265±0.049		
Ad-Lac Z	0.329±0.167^a^		
r-Ad-hIFN-λ1	0.772±0.075^b^	63.664	<0.001

a Ad-Lac Z group versus the blank group, P=0.334;

b r-Ad-hIFN-λ1 group versus the other two groups, P<0.001.

AI, apoptosis index; r-Ad-hIFN-λ1, human interferon-λ1 recombinant adenovirus; Ad-Lac Z, adenovirus encoding bacterial β-galactosidase.

**Table V:** Comparison of the NK cell proliferation in the spleen among the three groups.

Group	NK cell proliferation rate (%)	F-value	P-value
Blank	16.35±1.43		
Ad-Lac Z	17.70±1.09^a^		
r-Ad-hIFN-λ1	26.53±1.54^b^	162.96	<0.0001

a Ad-Lac Z group versus the blank group, P=0.065;

b r-Ad-hIFN-λ1 group versus the other two groups, P<0.0001.

NK, natural killer; r-Ad-hIFN-λ1, human interferon-λ1 recombinant adenovirus; Ad-Lac Z, adenovirus encoding bacterial β-galactosidase.
